# Public behavioral compliance challenges in household medical waste management: an evolutionary game perspective on policy

**DOI:** 10.3389/fpubh.2026.1812333

**Published:** 2026-04-15

**Authors:** Shuli Yang, Junwu Chai, Yang Yu

**Affiliations:** School of Economics and Management, University of Electronic Science and Technology of China, Chengdu, China

**Keywords:** household medical waste management, peer effect, policy design, public behavioral compliance, tripartite evolutionary game

## Abstract

Household medical waste (HMW) management faces significant challenges, including low waste recycling participation rates and gaps in policy regulatory. This study aims to promote a healthy living environment and wellbeing for all citizens by developing a tripartite evolutionary game model to examine the policy effectiveness mechanism and the strategic interactions among governments, medical waste disposal institutions and residents. Our findings are threefold. First, the analysis reveals a stable equilibrium (1, 1, 0), indicating that under loose government regulation, community residents and medical waste disposal institutions establish a code of conduct for compliant recycling practices, market-oriented cooperation is more sustainable than intensive government enforcement. This result can be explained by the peer effect: residents' non-compliant behavior generates feelings of guilt, and the increased psychological cost proves more effective than external policy intervention. This finding challengs the conventional assumptions that intensive government intervention achieves greater effectiveness. Second, through numerical analysis, we identify the thresholds at which key parameters become effective in government policy design. In particular, investment in environmental education exhibits a jump effect once it exceeds a critical threshold, enabling the system to bypass intermediate stages and move directly toward the optimal equilibrium. Third, we propose a three-phase household medical waste management strategy encompassing environmental education enhancement, institution's profitability optimization, and regulatory cost control to facilitate public smooth transition to optimal equilibrium.

## 1 Introduction

With the expansion of healthcare services and widespread use of household medical supplies, household medical waste (HMW), including discarded needles, expired pharmaceuticals, medical gauze, and diagnostic test kits, has increased annually. The COVID-19 pandemic dramatically increased stockpiled preventive materials (antigen kits, thermometers, medications, and N95 masks). Global medical waste generation has reached unprecedented levels, and the medical waste management market is projected to grow at a compound annual growth rate (CAGR) of 7.4% from 2025 to 2034 (1). Approximately 15% of healthcare waste is hazardous and can be infectious, toxic, or radioactive, improper disposal poses significant environmental pollution and public health risks.

Many countries have made substantial efforts in developing and implementing HMW recycling management policies. For example, the United States enacted the “Resource Conservation and Recovery Act” and the “Medical Waste Tracking Act,” substantially improving recycling rates through community collection points and cooperation with healthcare institutions (2). EU countries such as Germany and the Netherlands have achieved recycling rates of over 80% recycling rates through strict classification standards, economic incentives, and education. Germany's “Circular Economy Act” requires pharmacies to collect expired medications free of charge, achieving a compliance rate of over 70% (3). Korea's “Extended Producer Responsibility System” requires manufacturers to handle HMW recycling, significantly improving efficiency (4). These examples demonstrate that government regulation provides essential external constraints that are crucial for improving both public resident recycling compliance and institution's responsiveness. However, many developing countries still lack comprehensive HMW regulations. In India, although unused and expired antibiotics are classified as hazardous waste and should be collected separately from other household waste, these rules are not widely known or followed by the public. Brazil did not introduce regulations for collecting medicinal waste from residents until 2020 (5). In some regions, illegal medical waste reuse even occurs through informal channels (6). These examples demonstrate that developing countries are significantly lagging behind in formulating and implementing medical waste management regulations, highlighting the urgent need to regulate the chaotic state of medical waste through strengthened public policy development.

Furthermore, public non-compliance behavior also has a significant impact on the effectiveness of policy implementation. Non-compliance behavior refers to an individual's failure to adhere to established norms, policies, or social expectations, manifesting as rule deviation or refusal to comply (7). Residents' non-compliance significantly impedes the development of HMW management. Specifically, some residents fail to follow policy guidelines by not bringing HMW to designated collection stations. Residents' non-compliance with HMW recycling may stem from multiple factors. On the one hand, it may result from a lack of relevant knowledge (8). On the other hand, driven by cost considerations, residents may perceive the cost of complying with HMW recycling as outweighing their concern for social norms and environmental safety (9, 10).

Consequently, policy formulation and public compliance jointly influence policy effectiveness. With growing attention to the interaction between social context and individual behavior, many studies have examined how to enhance public compliance. A major line of inquiry centers on the peer effects. The tendency for individuals within the same group to exhibit similar behaviors is termed the peer effect, which significantly influences environmental behavior through the invisible pressure of social norms (11). When a group participates in recycling, individuals' willingness to do so also increases (12, 13). Recent empirical research further demonstrates that peer effects have a significant positive impact on residents' environmental behaviors (14). This suggests that the peer effect can serve as an effective social mechanism for addressing residents' non-compliance with HMW recycling. However, existing literature primarily focuses on household waste sorting and medical waste disposal by healthcare institutions, while paying limited attention to HMW, which poses risks to community public health. Research examining the interaction between policies and public compliance in this context is even more scarce. Therefore, this study argues that research in the field of HMW management is both urgent and necessary. Approaching the topic from the perspective of peer effects on HMW recycling behavior represents an innovative interdisciplinary approach.

Furthermore, the indispensable role of medical waste disposal institutions in HMW management should also be emphasized, as they provide the physical infrastructure, transportation, and convenience required for HMW recycling. Their primary function is to collect and dispose of medical waste generated by healthcare facilities in compliance with government policies. In countries without HMW management policies, medical waste disposal institutions typically do not proactively establish HMW recycling operations because collecting and disposing of HMW is often economically unfeasible due to its low volume and scattered distribution. Institutional theory research indicates that penalty policies compel firms to adopt compliant behavior under legitimacy pressure, whereas non-compliant actions are often performance-driven (15, 16). Even in countries that implemented HMW management policies, institutions' negative responses to HMW recycling policies still occur, attributed to high costs, insufficient market incentives, or “wait-and-see” attitudes. Therefore, the effectiveness of government regulatory policies in promoting institutional participation, whether through incentives or penalties, remains to be verified in the context of HMW recycling.

Motivated by these challenges, this study addresses the following questions: (1) What are evolutionarily stable strategies for HMW recycling management policies under tripartite stakeholder interactions? (2) What is the mechanism by which the peer effect influences public compliance behavior? (3) What measures can enhance the operational efficiency of environmental policy implementation?

To address these questions, we construct an evolutionary game model involving community residents, medical waste disposal institutions, and the government. Evolutionary game theory is preferred because it models dynamic strategy adaptation among heterogeneous stakeholders, captures how the strategies of various parties gradually evolve and tend toward equilibrium or disequilibrium as policies, costs, and incentives change. Empirical studies of stakeholder interactions in environmental supply chains demonstrate that evolutionary models identify stable strategy regions and critical thresholds, providing actionable guidance for policy design that static or purely empirical approaches cannot deliver (17). Through theoretical analysis, numerical simulation, and policy incentive-penalty mechanism modeling, this research provides theoretical support and practical guidance for improving HMW recycling systems.

This study innovatively extends the application of evolutionary game theory in HMW management by incorporating insights from behavioral economics, particularly the peer effect as a social mechanism influencing individual behavior. The combination of a tripartite evolutionary game and peer effect is necessary for this study for several reasons: first, traditional static game theory assumes fully rational actors with fixed strategies, which is unrealistic in environmental contexts because the social norms pressure often outweighs an individual's intrinsic economic motivation (18). In this study, both strategic equilibria among multiple stakeholders and social interaction under normative pressure are indispensable. Second, evolutionary game models can simulate not only whether the public will comply with HMW management policies, but also the social conditions under which compliance is likely to emerge. Peer effects drive chain reactions in public behavior, once compliance reaches a certain threshold, group behavior rapidly shifts in a positive direction, and vice versa (19). Combining these two approaches allows for the validation of key threshold effects. Third, many existing studies focus on enhancing public compliance, with most concentrating on a single mechanism (economic incentives or social norms). This study combines tripartite evolutionary game theory with the peer effect, thereby creating a dynamic framework that integrates multiple mechanisms. The integration of these two concepts can help policymakers design precise incentives tailored to multiple participants, determine appropriate investments to enhance the responsiveness of medical waste disposal institutions, maintain public support for the policy, and avoid reliance on purely punitive economic sanctions. It offers greater sustainability than traditional single-mechanism models. This study investigates sustainable HMW recycling systems through a comprehensive analysis of stakeholder interactions and policy mechanisms. Specifically, it examines how government regulatory frameworks, the operational strategies of medical waste disposal institutions, and residents' behavioral compliance jointly influence HMW management systems, thereby filling theoretical gaps and contributing to a healthier living environment and improved wellbeing for citizens.

This paper is organized as follows: Section 2 reviews relevant literature and theoretical frameworks; Section 3 introduces evolutionary game model assumptions and construction; Section 4 analyzes evolutionary equilibrium results and simulations; Section 5 discusses main conclusions and management recommendations.

## 2 Literature review

Medical waste management is a critical issue in public health and environmental protection. Our study is primarily associated with two research streams. The first stream focuses on factors influencing the formulation of medical waste recycling policies. The second stream examines the mechanisms affecting public compliance from a behavioral economics perspective.

### 2.1 The factors influencing formulation of medical waste recycling policies

The factors influencing medical waste recycling behavior are multi-dimensional. Some studies focus on knowledge and awareness factors, showing that individual knowledge of medical waste recycling and environmental awareness directly influence recycling behavior. Enhanced environmental awareness and training reduce improper disposal at sources (20). A lack of waste management knowledge exposes individuals and communities to health and environmental hazards (21). Environmental psychology and behavioral economics studies demonstrate that social norms, values, and economic incentives positively impact recycling behavior (22). Recent evidence further confirms that individual environmental awareness positively moderates the effect of social norms on pro-environmental behavior (18). A few studies focus on systemic infrastructure factors: recycling system infrastructure design represents a key determinant affecting participation rates. An analysis by Saphores and Nixon (23) of household recycling policies for metals, glass, and plastics find that infrastructure accessibility within recycling systems is a key driver of participation. Amirteimoori et al. (24) developed algorithms for configuring sustainable waste management systems to optimize facility construction, transportation system utilization, and operational decision-making. As for policy intervention factors: government policies or income levels can influence medical waste generation (25). Abila and Kantola (26) demonstrate that financial incentives promote consumer participation in waste disposal. Furthermore, the government intervention and corporate social responsibility effectively mitigate medical waste-related environmental pollution (27). Liu et al. (28) explored how government incentives can promote the development of formal channels for household pharmaceutical waste recycling and curb the growth of informal channels.

Although the aforementioned studies provide an important foundation for understanding and formulating medical waste recycling policies, several key limitations remain: first, existing research perspectives tend to be one-dimensional, lacking an integrated framework that accounts for multiple factors. For example, some studies focus on individual knowledge and attitudes, others emphasize economic incentives, and still others highlight social norms. However, actual public decision-making is often the result of the combined influence of knowledge, economic rationality, and social norms. This one-dimensional research perspective struggles to explain why different groups exhibit starkly different behavioral patterns under similar cost-benefit conditions. Accordingly, there is a need to construct a systematic theoretical framework that integrates the interactive roles of environmental knowledge, recycling system design, and policy interventions in order to address these gaps. Second, while existing empirical studies confirm the influence of social norms on environmental behavior, most rely on cross-sectional data designs and thus remain static in nature, lacking a dynamic evolutionary perspective. Consequently, they fail to reveal how new norms form, diffuse, and evolve in the context of multiple stakeholder participation (government, waste disposal institutions, and residents). Third, the majority of research is confined to specific regions or healthcare institutions, with insufficient research on household-generated medical waste outside healthcare settings, representing a significant research gap. This gap leaves the direct applicability of existing research findings to the context of household medical waste open to further verification. To address these gaps, this study focuses on the HMW, an area that remains largely underexplored. We construct a comprehensive tripartite evolutionary game framework that simultaneously models three key mechanisms: the incentive and constraint mechanisms of government policies, the behavioral interaction mechanisms of community residents, and the strategic influence mechanisms of medical waste disposal institutions. This framework allows us to explore the dynamic equilibrium evolution among the three parties.

### 2.2 Public non-compliance

From a behavioral economics perspective, social contextual factors significantly influence individual decision-making processes and behavioral outcomes. Community residents' non-compliance behavior causes HMW treatment development stagnation. Non-compliance behavior refers to individuals' failure to follow established norms, policies, or social expectations, which may manifest as rule deviation or refusal to comply (7). Compliance behavior represents the opposite. Public non-compliance often stems from cost considerations, questioning of policy legitimacy, or indifference (10, 16).

In the context of medical waste recycling, improving public compliance is a key issue that many studies have sought to address. A key theoretical premise is that an individual's decision to join a collective action depends on the number of participants already engaged (12). This study is the first to systematically formalize social interaction into a testable mathematical model, revealing how micro-level individual decisions aggregate into macro-level collective behavior. Some experimental studies show that recycling behavior depends largely on perceptions of others' behavior, and that group identity increases an individual's contribution to recycling (13). Another important theoretical foundation is the peer effect. Manski (11) classifies peer effects into three categories: endogenous effects (individual behavior influenced by group behavior), exogenous effects (individual behavior influenced by group characteristics), and associative effects (similar individuals exhibiting similar behavior due to shared environments), which serve as a framework for explaining the social diffusion mechanisms of environmental behavior. Recent empirical studies indicate that peer effects have a significant positive impact on residents' environmental behaviors (14). Granovetter's threshold model and Manski's peer influence framework offer complementary theoretical perspectives. Recent research combining the two has demonstrated the threshold effect of peer influence in driving collective behavior, showing that group behavior shifts rapidly once a threshold is crossed (19).

Although existing evidence reveals the mechanisms of peer effects and the existence of threshold effects, most studies rely on static or unidirectional diffusion models that fail to incorporate multi-stakeholder strategic interactions. In other words, while prior research explains how public compliance spreads, it does not address how policies should be designed to trigger such diffusion. Especially in the field of HMW management, the strategic adjustments and dynamic equilibrium processes among governments, residents, and medical waste disposal institutions remain underexplored. To address these gaps, this study innovatively integrates tripartite evolutionary game theory with peer effects to address multi-stakeholder dynamics, simulate threshold-driven behavioral shifts, and design sustainable policy mechanisms, thereby filling theoretical gaps in HMW management, and providing theoretical support for policy formulation and public health safety.

## 3 Methods

This study constructs a tripartite evolutionary game model to examine the strategic interactions among the government, medical waste disposal institutions, and residents through theoretical analysis and numerical simulation. The model involves three types of stakeholders: residents, medical waste disposal institutions, and the government. The behavioral strategy set for residents is (compliance, non-compliance), the behavioral strategy set for medical waste disposal institutions is (active response, negative response), and the behavioral strategy set for the government is (strict supervision, loose supervision). The specific meaning of each strategy will be detailed under the following assumptions. Since the three stakeholders choose strategies based on their own interests, and since each party's strategic choice is influenced by the other two parties, none of them can be assumed to make fully rational decisions. Therefore, all three are treated as boundedly rational players.

### 3.1 Model assumptions

Assumption 1 Government supervision of medical waste disposal institutions is divided into strict supervision and loose supervision. Strict supervision (with probability *z*, where *z*∈(0, 1)) means that the government provides incentives to medical waste disposal institutions that actively respond to the HMW recycling policy and imposes penalties on those that respond negatively. Loose supervision (with probability 1−*z*) involves neither incentives nor penalties. Medical waste disposal institutions' active response to government regulations means adding disposal facilities and services for HMW (with probability *y*, where *y*∈(0, 1)); negative response means not adding disposal facilities and services for HMW (with probability 1-y). Resident compliance (with probability *x*, where *x*∈(0, 1)) means residents comply with government advocacy and conduct designated classified recycling of HMW; non-compliance (with probability 1−*x*) means residents do not comply with government advocacy for designated classified recycling of HMW.

Assumption 2 When the government implements strict supervision strategies for medical waste disposal institutions, it needs to invest regulatory governance costs *D*, including fixed expenditures for human resources and technology; implementing loose supervision strategies requires no regulatory governance costs. Research shows that public environmental education campaigns can significantly impact pharmaceutical waste management (29). Therefore, when the government implements strict supervision strategies, it also needs to conduct environmental education for residents, investing environmental education costs *W*, advocating the recycling of HMW to designated locations, which is then processed harmlessly by professional medical waste disposal institutions. When residents comply with HMW recycling and medical waste disposal institutions actively respond to government regulations, positive social outcomes are generated, and the government obtains social benefits *S*, which may include implicit environmental values. When residents do not comply with HMW recycling and medical waste disposal institutions respond negatively to government regulations, negative social outcomes are generated, and the government faces higher-level administrative penalties, resulting in environmental accountability losses *T*.

Assumption 3 Residents are the generators of HMW and are responsible for its disposal. When residents comply with government advocacy for designated classified recycling of HMW, they incur time and physical effort costs *C*_*C*_ and obtain perceived benefits *V* after recycling. Resident perceived benefits mainly come from self-protection against health and safety hazards, and achievement or satisfaction from participating in environmental behaviors. Additionally, when the government conducts environmental education for residents, resident compliance perceived benefits increase with environmental education, i.e., *V* = *V*(1+*W*). When residents do not comply with government advocacy for such classified disposal, they face health and safety risks and incur psychological costs *C*_*NC*_ such as inner guilt from not participating in environmental behaviors.

Generally, the explicit costs (time and physical effort) of compliant recycling behavior are higher than the psychological costs of non-compliant recycling behavior, as recycling requires more effort than non-recycling, i.e., _*C*_*C*_>*CNC*_. However, due to peer effects, when more residents comply with recycling (*x*>0.5), individuals find recycling behavior convenient when supported by social norms (30). Non-compliant residents experience greater guilt and psychological pressure, making non-compliant recycling require more effort than compliant recycling, i.e., _*C*_*C*_<*CNC*_ when *x*>0.5. Rationale for setting the threshold at 0.5: theoretical critical models suggest that when the driving force exceeds 25% of the group, new social norms will emerge, prompting the majority to rapidly adopt the new norm (31). However, the emergence of social norms alone cannot exert pressure on residents who fail to comply with environmental regulations. Therefore, this study adopts a threshold of 50%. Once the probability of resident compliance reaches 50%, the norm becomes the mainstream social expectation, thereby generating stronger social pressure and increasing the psychological cost of non-compliance. This aligns with traditional economic assumptions, where individual decisions during behavioral shifts depend on group expectations, and the 50% threshold represents an equilibrium and stable turning point (32).

Additionally, we set β as the moderating coefficient of residents' perceived recycling costs influenced by recycling facility accessibility, where β∈(0, 1). Under waste disposal institutions' negative response behavior, recycling costs become *C*_*C*_(1+β), meaning that institutions have not established nearby community recycling facilities and services for residents, who must instead go to medical institutions for recycling. Therefore, residents need to pay higher recycling costs due to reduce convenience. Correspondingly, *C*_*NC*_(1−β) indicates that residents attribute their non-compliant recycling behavior to inconvenient recycling, thereby reducing psychological costs. In our context, residents update their HMW recycling compliance behavior by observing neighboring residents' behaviors and related benefits, which is a dynamic learning process of peer interaction, where each individual attempts to maximize their expected benefits over time.

Assumption 4 Medical waste disposal institutions have fixed original disposal business income *R* from healthcare facilities. When actively responding to government regulations, the cost for adding HMW disposal services is *I*, the income is *R*′. Since HMW disposal volume is usually smaller than the medical waste disposal volume from healthcare facilities in their original business, the income satisfies *I*<*R*′ < *R*. Under government strict supervision strategies, medical waste disposal institutions' active response strategies also receive government rewards *M*, while negative response strategies are penalized by the government with fines *F*.

**Figure 1:**
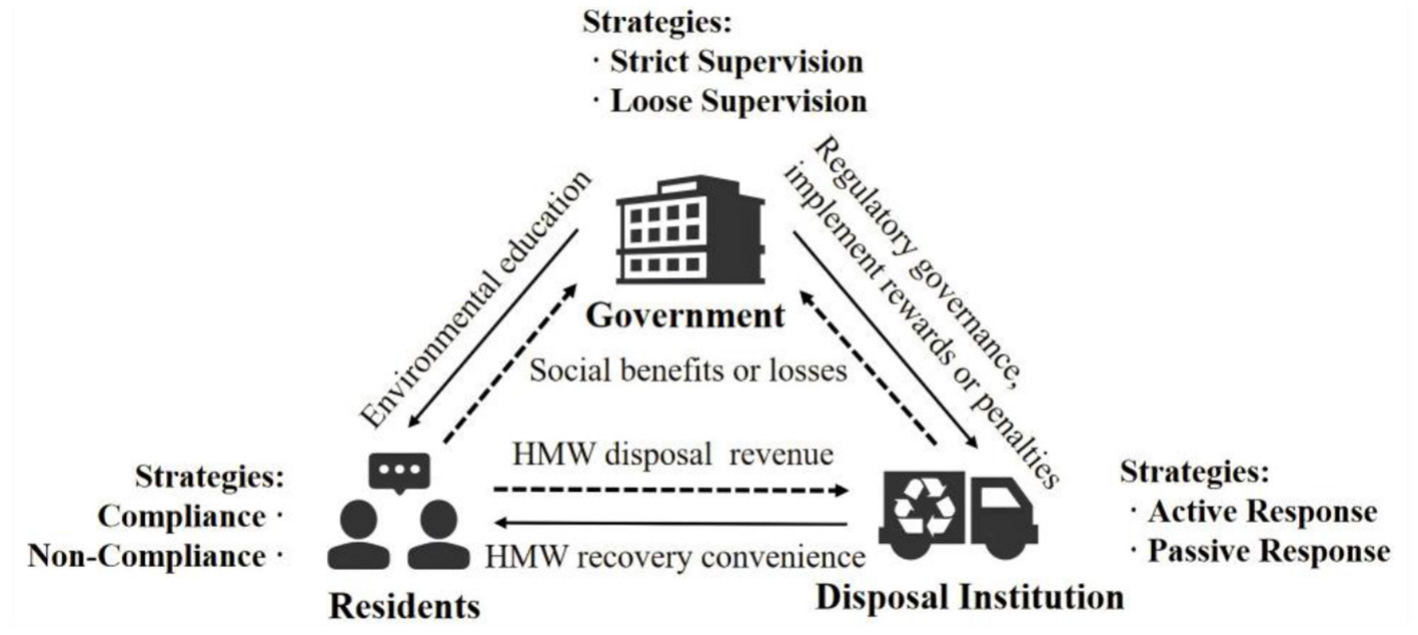
Game relationships among three stakeholders.

The relevant parameter symbols and meanings are shown in Table 1, and the game relationships among the three stakeholders are shown in Figure 1.

**Table 1:** Parameter symbols and explanations.

Participants	Parameter	Explanations
Government	*D*	Supervision costs incurred by government for institutions
	*W*	Environmental education costs invested by government for residents
	*S*	Social benefits obtained by government from effective HMW management
	*T*	Environmental accountability losses incurred by government
Residents	*V*	Perceived benefits obtained by residents from compliant recycling behavior
	*C*_*C*_	Compliance costs incurred by residents for HMW recycling
	*C*_*NC*_	Psychological costs experienced by residents for non-compliant behavior
	β	Moderating coefficient of facility accessibility on resident' perceived recycling costs
Medical waste disposal institutions	*R*	Institution's existing disposal business revenue
	*R*′	Institution's additional HMW disposal business revenue
	*I*	Institution's additional HMW disposal business costs
	*M*	Rewards provided by government for institutions' active response
	*F*	Penalties imposed by government for institutions' negative response

### 3.2 Modeling

Based on the aforementioned assumptions, the evolutionary game payoff matrix for residents, medical waste disposal agencies, and the government is presented in Table 2.

**Table 2:** The payoff matrix of the tripartite decision.

Stakeholders (*f, m, g*)	Government (*g*) strict supervision		Government loose supervision	
	Institution (*m*) active response	Institution negative response	Institution active response	Institution negative response
Resident (*f*) compliance	*V*(1+*W*)−*C*_*C*_	*V*(1+*W*)−(1+β)*C*_*C*_	*V*−*C*_*C*_	*V*−(1+β)*C*_*C*_
	*R*+*R*′−*I*+*M*	*R*−*F*	*R*+*R*′−*I*	*R*
	*S*−*D*−*W*−*M*	*F*−*D*−*W*	*S*	0
Resident non-compliance	−*C*_*NC*_	−(1−β)*C*_*NC*_	−*C*_*NC*_	−(1−β)*C*_*NC*_
	*R*−*I*+*M*	*R*−*F*	*R*−*I*	*R*
	−*D*−*W*−*M*	*F*−*D*−*W*−*T*	0	−*T*

## 4 Evolutionary equilibrium analysis

### 4.1 Replicator dynamics equations

Based on the payoff matrix in Table 2 and relevant evolutionary game theory, the expected payoffs and replicator dynamics equations for each stakeholder can be derived. We define the expected payoffs and average payoff for residents engaging in compliant HMW recycling and non-compliant HMW recycling as *E*_*fC*_, *E*_*fNC*_, and $\bar{E_{f}}$, respectively, see Equations 1–3. The expected and average payoffs for medical waste disposal institutions that actively respond to government regulations and those that negatively respond to government regulations are denoted as *E*_*mI*_, *E*_*m*0_, and $\bar{E_{m}}$, respectively, see Equations 4–6. The expected and average payoffs for the government implementing strict supervision and loose supervision of medical waste disposal institutions are denoted as *E*_*gD*_, *E*_*g*0_, and $\bar{E_{g}}$, respectively, see Equations 7–9.


$$\begin{array}{l} E_{fC}=yz\left({V\left(1+W\right)-C}_{C}\right)+y\left(1-z\right)\left({V-C}_{C}\right)\\ \;+\left(1-y\right)z\left({V\left(1+W\right)-\left(1+\beta \right)C}_{C}\right)\\ \;-\left(1-y\right)\left(1-z\right)\left({V-\left(1+\beta \right)C}_{C}\right) \end{array}$$ (1)

$$\begin{array}{l} E_{fNC}=-C_{NC}(yz+y(1-z))+((1-y)z+(1-y)(1-z))\\ \;(-(1-\beta )C_{NC})=-C_{NC}y-(1-\beta )C_{NC}(1-y) \end{array}$$ (2)

$$\begin{array}{c} \bar{E_{f}}=xE_{fC}+\left(1-x\right)E_{fNC} \end{array}$$ (3)

$$\begin{array}{l} E_{mI}=xz\left(R+R^{\prime }-I+M\right)+x\left(1-z\right)\left(R+R^{\prime }-I\right)\\ \;+\left(1-x\right)z\left(R-I+M\right)\\ \;+\left(1-x\right)\left(1-z\right)\left(R-I\right) \end{array}$$ (4)

$$\begin{array}{l} E_{m0}=xz\left(R-F\right)+x\left(1-z\right)R+\left(1-x\right)z\left(R-F\right)\\ \;+\left(1-x\right)\left(1-z\right)R \end{array}$$ (5)

$$\begin{array}{c} \bar{E_{m}}=yE_{mI}+\left(1-y\right)E_{m0} \end{array}$$ (6)

$$\begin{array}{l} E_{gD}=xy\left(S-D-W-M\right)+x\left(1-y\right)\left(F-D-W\right)\\ \;+y\left(1-x\right)\left(-D-W-M\right)\\ \;+\left(1-x\right)\left(1-y\right)\left(F-D-W-T\right) \end{array}$$ (7)

$$\begin{array}{c} E_{g0}=xyS-T\left(1-x\right)\left(1-y\right) \end{array}$$ (8)

$$\begin{array}{c} \bar{E_{g}}=zE_{gD}+{\left(1-z\right)E}_{g0} \end{array}$$ (9)

Based on this, according to the principles of tripartite evolutionary games, the replication dynamics equations for relevant stakeholders derived from Equations 1–9 are as follows (Equation 10–12):


$$\begin{array}{l} F\left(x\right)=\frac{d_{x}}{d_{t}}=x\left(E_{fC}-\bar{E_{f}}\right)=x\left(1-x\right)(C_{NC}+V(-1-2y(-1\\ \;+z)+\left(2+W\right)z)+C_{NC}\left(-1+y\right)\beta +C_{C}(-((-1\\ \;+2z)\left(1+\beta \right))+y\left(-2-\beta +2z\left(1+\beta \right)\right))) \end{array}$$ (10)

$$F\left(y\right)\;=\frac{d_{y}}{d_{t}}=y\left(E_{mI}-\bar{E_{m}}\right)=y(1-y)(xR^{\prime }-I+z(M+F))$$ (11)

$$F\left(z\right)\;=\frac{d_{z}}{d_{t}}=z\left(E_{gD}-\bar{E_{g}}\right)=z\left(1-z\right)(\left(1-y\right)F-D-W-yM)$$ (12)

### 4.2 Stability analysis

Based on the above analysis, a three-dimensional dynamic system can be obtained from *F*(*x*), *F*(*y*), and *F*(*z*), where the replicator dynamic equations of the three stakeholders are set to zero, i.e., $\frac{d_{x}}{d_{t}}=0$, $\frac{d_{y}}{d_{t}}=0$, $\frac{d_{z}}{d_{t}}=0$, yielding eight boundary equilibrium points: (1, 0, 0), (1, 1, 0), (1, 0, 1), (1, 1, 1), (0, 0, 0), (0, 0, 1), (0, 1, 0), (0, 1, 1).

The existence condition for interior equilibrium points is 0 < x^*^ < 1, 0 < y^*^ < 1, 0 < z^*^ < 1, yielding the following constraint conditions: *F*>*D*+*W*, *D*+*W*+*M*>0, $\frac{\left(I-R^{\prime }\right)}{F+M}<z<\frac{I}{F+M}$, *VW*>0, _*C*_*C*_>*CNC*_ (*x*>0.5). Following the evolutionary equilibrium strategy assessment method proposed by Friedman (33), the Jacobian matrix (Equation 13) is obtained based on the replicator dynamic Equations 10–12. Substituting the above eight equilibrium points into the Jacobian matrix yields the corresponding eigenvalues, as shown in Table 3.


$$J=\left[\begin{array}{ccc} \frac{\partial F\left(x\right)}{\partial x} & \frac{\partial F\left(x\right)}{\partial y} & \frac{\partial F\left(x\right)}{\partial z}\\ \frac{\partial F\left(y\right)}{\partial x} & \frac{\partial F\left(y\right)}{\partial y} & \frac{\partial F\left(y\right)}{\partial z}\\ \frac{\partial F\left(z\right)}{\partial x} & \frac{\partial F\left(z\right)}{\partial y} & \frac{\partial F\left(z\right)}{\partial z} \end{array}\right]=\left[\begin{array}{ccc} u_{11} & u_{12} & u_{13}\\ u_{21} & u_{22} & u_{23}\\ u_{31} & u_{32} & u_{33} \end{array}\right]$$ (13)

**Table 3:** Eigenvalues of each equilibrium point.

Equilibrium point	Eigenvalues λ_1_	Eigenvalues λ_2_	Eigenvalues λ_3_
(0, 0, 0)	*C*_*NC*_(1−β)−*V*+*C*_*C*_(1+β)	−*I*	*F*−*D*−*W*
(1, 0, 0)	*C*_*NC*_(β−1)+*V*−*C*_*C*_(1+β)	*R*′−*I*	*F*−*D*−*W*
(0, 1, 0)	*V*+_*C*_*NC*_−*CC*_	*I*	−*D*−*M*−*W*
(0, 0, 1)	*V*(1+*W*)+*C*_*NC*_(1−β)−*C*_*C*_(1+β)	*M*+*F*−*I*	*D*+*W*−*F*
(1, 1, 0)	*C*_*C*_−*C*_*NC*_−*V*	*I*−*R*′	−*D*−*M*−*W*
(1, 0, 1)	_*C*_*C*_(1+β)−*CNC*_(1−β)−*V*(1+*W*)	*F*−*I*+*M*+*R*′	*D*−*F*+*W*
(0, 1, 1)	*C*_*NC*_+*V*(1+*W*)−*C*_*C*_	*I*−*M*−*F*	*D*+*M*+*W*
(1, 1, 1)	*C*_*C*_−*V*(1+*W*)−*C*_*NC*_	*I*−*M*−*F*−*R*′	*D*+*M*+*W*

In which:


$$\begin{array}{l} u_{11}=\;(1-2x)(C_{NC}+V(-1-2y(-1+z)+(2+W)z)\\ \;+C_{NC}(-1+y)\beta +C_{C}((1-2z)(1+\beta ))\\ \;+y(2+\beta -2z(1+\;\beta ))),\\ u_{12}=(1-x)x(-2V(z-1)+C_{NC}\beta +C_{C}(2z(1+\beta )-2-\;\beta )),\\ u_{13}=(1-x)x(V(2+W-2y)+2C_{C}(-1+y)(1+\;\beta )),\\ u_{21}=(1-\;y)yR^{\prime },\\ u_{22}=(2y-1)(I-(F+M)z-\;xR^{\prime }),\\ u_{23}=(F+M)(1-\;y)y,\\ u_{31}=\;0,\\ u_{32}=\;(M+F)\;z(z-\;1),\\ u_{33}=(D+W+F(y-1)+My)(2z-\;1), \end{array}$$

The evolutionary game equilibrium is influenced by multiple factors, and stable strategy equilibrium points differ under different factor combinations. For each equilibrium point, it represents an Evolutionarily Stable Strategy (ESS) only when all eigenvalues satisfy _*R*_*e*_(λ*i*_) < 0. Based on the signs of the real parts of the eigenvalues reported in Table 3, we conduct a stability analysis of the equilibrium points, and the detailed results are presented in Table 4.

**Table 4:** Local stability analysis of equilibrium points.

Equilibrium point	Eigenvalues	Stability type
(0, 0, 0)	*C*_*NC*_(1−β)−*V*+*C*_*C*_(1+β)>0, −*I* < 0, *F*−*D*−*W*⊗	Saddle point
(1, 0, 0)	*C*_*NC*_(β−1)+*V*−*C*_*C*_(1+β)⊗, *R*′−*I*⊗, *F*−*D*−*W*⊗	Saddle point/Unstable point
(0, 1, 0)	*V*+_*C*_*NC*_−*CC*_⊗, *I*>0, −*D*−*M*−*W* < 0	Saddle point
(0, 0, 1)	*V*(1+*W*)+*C*_*NC*_(1−β)−*C*_*C*_(1+β)⊗, *M*+*F*−*I*⊗, *D*+*W*−*F*>0	Saddle point/Unstable point
(1, 1, 0)	*C*_*C*_<*C*_*NC*_+*V*, *I*>*R*′, *D*+*M*+*W*>0	Stable point
(1, 0, 1)	_*C*_*C*_(1+β)−*CNC*_(1−β)−*V*(1+*W*)⊗, *F*−*I*+*M*+*R*′⊗, *D*−*F*+*W*⊗	Saddle Point/Unstable Point
(0, 1, 1)	*C*_*NC*_+*V*(1+*W*)−*C*_*C*_⊗, *I*−*M*−*F*⊗, *D*+*M*+*W*>0	Unstable Point
(1, 1, 1)	*C*_*C*_−*V*(1+*W*)−*C*_*NC*_⊗, *I*−*M*−*F*−*R*′⊗, *D*+*M*+*W*>0	Unstable Point

Where ⊗ indicates that the eigenvalues cannot be determined.

Scenario 1 Although (1, 1, 1) represents ideal comprehensive cooperation, eigenvalue analysis shows that it is not a stable point. Since *D* (regulatory costs), *M* (rewards), and *W* (education costs) are positive, eigenvalues *D*+*M*+*W*>0 always hold, making “comprehensive tripartite cooperation” mathematically impossible for ESS stability. Slight increases in regulatory intensity *z* decrease system returns, indicating that excessive regulation generates negative utility and undermines stability. Under *z* = 1 (complete strict supervision), the government faces excessive marginal regulatory costs where additional regulatory intensity costs exceed benefits. Excessive intervention crowds out market-based self-regulation, and the cumulative effect of the three types of costs creates an unsustainable financial burden for the government.

Scenario 2 When *C*_*C*_>*C*_*NC*_+*V*, *I*>*R*′, the evolutionarily stable strategy equilibrium point is (1, 1, 0): resident compliant recycling, institution active response, and government loose supervision. Residents choose compliance because high group recycling rates make compliance easier than non-compliance (*C*_*C*_<*C*_*NC*_), with perceived disposal benefits covering cost differences. Medical waste disposal institutions choose active response as benefits cover costs, improving processing efficiency and reducing pollution. The system reaches stability through proactive resident-institution cooperation. Stability analysis of (1, 1, 0) shows that “loose supervision” (*z* = 0) is more stable than “strict supervision” (*z* = 1). This suggests that market-driven cooperation is more sustainable than direct government intervention, with the effects of social norms outweighing those of administrative deterrence and thereby improving disposal efficiency. This suggests repositioning policy objectives from pursuing (1, 1, 1) to maintaining (1, 1, 0), transforming government from “strict regulator” to “limited facilitator,” supporting “light incentive-penalty regulation, heavy environmental education guidance” philosophy.

### 4.3 Numerical simulation analysis

#### 4.3.1 Parameter setting

This study establishes the following parameter settings of *V* = 8, *W* = 0.4, β = 0.3, *I* = 0.4, *R* = 100, *R*′ = 20, *F* = 8, *D* = 6, *M* = 2, *S* = 25, *T* = 15, when *x* = 0.75, *C*_*C*_ = 8, *C*_*NC*_ = 10, when *x* = 0.25, *C*_*C*_ = 12, *C*_*NC*_ = 10. The parameter configuration adheres to three principles: (1) parameter values must conform to realistic economic significance; (2) relative relationships between parameters must maintain rationality; (3) parameter settings must ensure the existence and stability of model solutions. All subsequent parameter configurations follow these principles. To ensure model robustness, sensitivity tests are conducted on critical parameters that significantly influence system evolution through ±20% perturbation analysis, examining variations in equilibrium point stability. Table 5 presents the eigenvalue analysis and stability assessment of boundary equilibrium points to validate the rationality of the baseline parameter settings.

**Table 5:** Eigenvalue analysis and stability assessment of boundary equilibrium points.

Equilibrium points	*C*_*C*_>*C*_*NC*_			*C*_*C*_<*C*_*NC*_			Practical implications
	λ*_1_*	λ*_2_*	λ*_3_*	$\lambda_{1^{\prime }}$	$\lambda_{2^{\prime }}$	$\lambda_{3^{\prime }}$	
(0, 0, 0)	11.4	−18	1.6	6.2	−18	1.6	Initial state
(1, 0, 0)	−11.4	2	1.6	−6.2	2	1.6	Unsustainable transitional state
(0, 1, 0)	6	18	−8.4	10	18	−8.4	Lack of resident cooperation
(0, 0, 1)	2.6	−8	−1.6	7.8	−8	−1.6	Limitations of a purely regulatory approach
(1, 1, 0)	−9.2	−2	−8.4	−13.2	−2	−8.4	Stable state of market-based collaboration
(1, 0, 1)	−2.6	12	−1.6	−7.8	12	−1.6	Lack of institutions
(0, 1, 1)	9.2	8	8.4	13.2	8	8.4	Overlook resident initiative
(1, 1, 1)	−9.2	−12	8.4	−13.2	−12	8.4	Diseconomies resulting from over-regulation

#### 4.3.2 The influence mechanism of peer effects

In accordance with Assumption 3, under peer effects, when majority choose compliant recycling (*x*>0.5), positive demonstration effects drive system convergence toward *x* = 1 (universal compliant recycling), non-compliant residents experience heightened psychological pressure, thereby making non-compliant recycling more effortful (_*C*_*C*_<*CNC*_). This can be explained by behavioral experiments showing that when residents perceive that the majority have adopted environmentally responsible behavior, their decision-making paradigm shifts from “cost minimization” to “identity maintenance” (34). When majority choose non-compliant recycling (*x* < 0.5), negative conformity drives the system convergence toward *x* = 0 (universal non-compliant recycling). Group non-compliance weakens individual responsibility through “law does not punish masses” mentality, reducing non-compliance psychological costs (*C*_*NC*_<*C*_*C*_), while minority compliance causes low facility utilization, creating societal vicious cycles. When *x* = 0.5, similar costs (*C*_*NC*_≈ *C*_*C*_) make behavior susceptible to random factors, leaving systems unstable.

Furthermore, the numerical variation of the eigenvalues in Table 5 indicates that the influence of peer effects is systematic and stable. By comparing the eigenvalues of the states (0, 0, 0) and (1, 1, 0), we can observe that λ_1_ = 24.6, ($\lambda_{1}=\lambda_{1}-\lambda_{1}^{\prime }$); comparing the eigenvalues of the states (0, 1, 0) and (1, 1, 0), we can observe that λ_1_ = 19.2; comparing the eigenvalues of the states (0, 0, 1) and (1, 1, 0), we can observe that λ_1_ = 15.8; comparing the eigenvalues of the states (0, 1, 1) and (1, 1, 0), we can observe that λ_1_ = 26.4. The enormous magnitude of the difference in eigenvalues between the left and right sides of the peer effect threshold ($\lambda_{1}vs.\lambda_{1}^{\prime }$) demonstrates that the great deviation in resident compliance. This provides quantitative evidence for the robustness of the peer effect hypothesis, and reveals the mechanism by which peer effects influence community residents' compliance with HMW recycling by affecting endogenous psychological costs.

Therefore, we derive several policy implications focused on leveraging peer effects: (1) initial “ice-breaking”: in *x* < 0.5 regions, use mandatory regulation or incentives to increase the number of initial compliers, enabling *x* to cross the 0.5 critical point through community worker demonstrations and social network diffusion. (2) Mid-term reinforcement: establish compliance rate disclosure systems (e.g., household recycling leaderboards) that utilizing peer competition psychology, while optimizing facility convenience to create market-driven and sustainable cycles with high compliance, system efficiency, and lower costs. (3) Long-term maintenance: embed compliant recycling into social norms through education, making it mainstream behavior while amplifying non-compliance costs and transforming perceived benefits into intrinsic moral satisfaction.

#### 4.3.3 Evolutionary trajectory and phase diagram analysis

**Figure 2:**
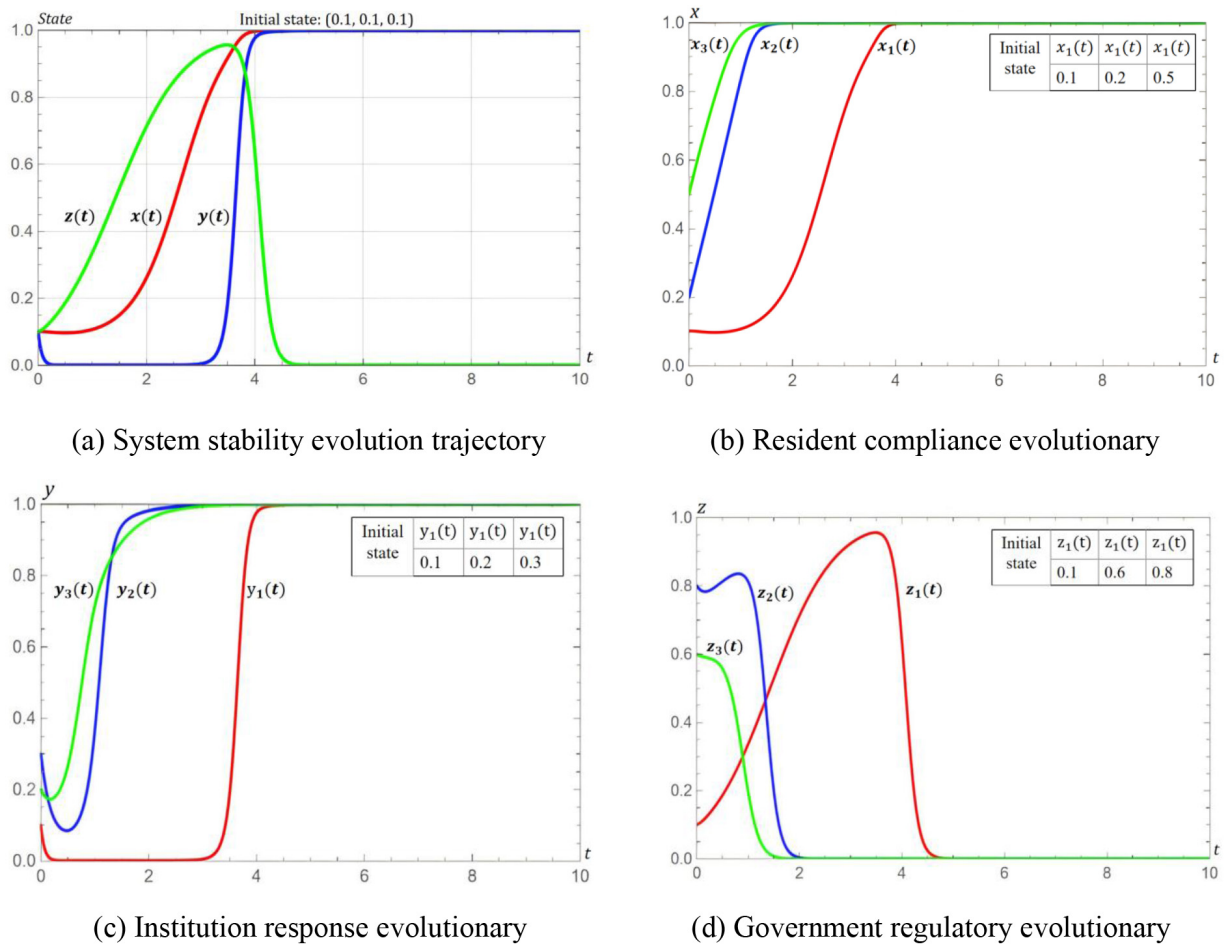
Evolutionary trajectories. **(a)** System stability evolution trajectory. **(b)** Resident compliance evolutionary. **(c)** Institution response evolutionary. **(d)** Government regulatory evolutionary.

The evolutionary trajectory simulation results are presented in Figure 2, showing that regardless of initial conditions, all evolutionary paths ultimately converge to point (1, 1, 0), thereby confirming the convergence and global stability of this equilibrium. Additionally, the z-coordinate (government regulatory intensity) exhibits the fastest convergence rate. This indicates that regulation quickly becomes redundant in the dynamic process, because once resident compliance and institutional engagement are activated, spontaneous mechanisms begin to replace mandatory oversight.

The analysis validates a dynamic evolutionary pathway: (0, 0, 0) → (1, 0, 0) → (1, 1, 0), which is consistent with the policy logic of residents leading, institutions following, and government moderating. Our research reveals a novel theoretical framework where the optimal regulatory strategy includes initial intervention, interim guidance and long-term withdrawal, diverging from the traditional static or rigid continuous regulation models. Traditional environmental regulatory systems often exhibit limitations due to their static and rigid nature (35, 36). Once regulations are established, they become entrenched over the long term due to path dependence (37) and institutional inertia, making it difficult to adapt to rapidly changing technological and economic environments. This framework provides significant guidance for environmental governance, market regulation, and social management in practice.

**Figure 3:**
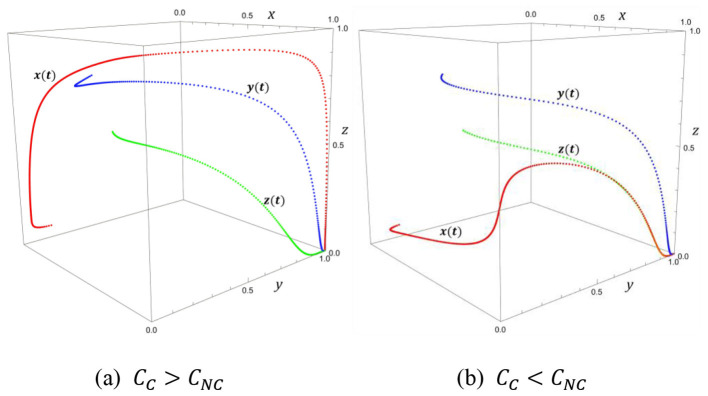
Three-dimensional phase map. **(a)** *C*_*C*_>*C*_*NC*_. **(b)** *C*_*C*_<*C*_*NC*_.

The three-dimensional phase diagram analysis reveals fundamental patterns within complex governance systems. The point (1, 1, 0) exhibits a substantial basin of attraction (see Figure 3), regardless of initial governance conditions, the system ultimately converges to this optimal state. Consistent with the evolutionary trajectory analysis, the phase diagram confirms that regulatory intensity is the most sensitive policy variable, even minor regulatory adjustments generate amplified effects. Therefore, policy implementation requires patience, allowing moderate oscillations while avoiding frequent adjustments due to short-term fluctuations. Regulatory adjustments should be gentle rather than aggressive, while appropriately relying on the system's inherent self-correction capabilities.

### 4.4 Parameter sensitivity analysis

#### 4.4.1 Impact of environmental education investment

The analysis of parameter *W* variations within the range of 0.1–1 yields results presented in Table 6. The environmental education investment parameter analysis reveals the critical role of education in multi-party evolutionary games and optimal allocation principles. We find that increases in environmental education investment significantly enhance system evolution speed, primarily because educational investment elevates residents' environmental perception benefits *V* = *V*(1+*W*), thereby altering the expected payoff structure of all stakeholders and accelerating convergence toward optimal equilibrium. When the environmental education investment is greater (*W*>0.5), the system can bypass the intermediate state (1, 0, 0) and evolve directly from (0, 0, 0) to (1, 1, 0). The theoretical mechanism underlying this phenomenon is that sufficiently high environmental education investment raises residents' environmental perception benefits to a level that simultaneously stimulates both resident compliance and institutional engagement at the early stage of the game. As a result, the system avoids suboptimal equilibrium traps that may arise from unilateral action.

**Table 6:** Impact of environmental education effects on system stability.

W	V	Stability			Convergence time
		(0, 0, 0)	(1, 0, 0)	(1, 1, 0)	
0.1	8.8	Unstable	Stable	Stable	45
0.3	10.4	Unstable	Stable	Stable	28
0.5	12.0	Unstable	Stable	Stable	18
0.7	13.6	Unstable	Unstable	Stable	12
0.9	15.2	Unstable	Unstable	Stable	8

From a policy design perspective, this finding provides crucial guidance for education investment strategies in environmental governance. First, strategic education investment is key to achieving leapfrog transformation in governance models, thereby helping systems avoid inefficient evolutionary pathways. Second, education investment should be balanced with other policy instruments (such as incentive mechanisms and regulatory intensity), as excessive reliance on a single policy tool may prove counterproductive. Policymakers must determine optimal education investment levels based on cost-benefit analysis to achieve effective policy resource allocation.

#### 4.4.2 Impact of institution profitability

By adjusting the ratio of revenue *I* to cost *R*′ parameters, this study analyzes the influence of medical waste disposal institution profitability on system stability, as presented in Table 7. We demonstrate that maintaining a profitable revenue-cost ratio constitutes a necessary condition *R*′/*I* >1 for sustaining the stable equilibrium (1, 1, 0). When new HMW disposal business revenue cannot cover corresponding costs, the rational choice for institutions is to exit active participation, causing the system to deviate from the optimal equilibrium state and revert to a suboptimal mode requiring mandatory government regulation. Furthermore, the determination of the optimal ratio *R*′/*I* interval of 1.05–1.15 embodies the importance of the moderate profitability principle in multi-party games. From an evolutionary game perspective, moderate profit levels ensure the stability of institution's strategy selection, institutions will neither easily abandon cooperation due to meager returns nor adopt opportunistic behavior due to excessive profit temptation, thereby maintaining the long-term equilibrium state of the system. Notably, the deeper implication of this research finding lies in revealing the potential threat of excessive profit-seeking to system stability. When the ratio *R*′/*I* exceeds the upper limit of 1.15, excessively high profit levels may lead to distorted institution's behavior, manifested as the excessive pursuit of short-term interests, potentially triggering countermeasures from other stakeholders (particularly residents and the government). Under such conditions, the system may transition from stable cooperative equilibrium to unstable competitive or confrontational states, ultimately damaging the long-term interests of all participants. From a policy design perspective, policymakers need to thoroughly understand the true cost structure of institutions, reasonably set revenue expectations, and ensure that incentive levels can fully mobilize institution enthusiasm without triggering moral hazard or social inequity issues.

**Table 7:** Impact of institution profitability parameters on (1, 1, 0) stability.

*R*^′^/*I*	λ_1_	λ_2_	λ_3_	Stability	Implications
0.9	−9.2	1.8	−8.4	Unstable	Insufficient profitability, low participation enthusiasm
1.0	−9.2	0.0	−8.4	Marginal stable	Break-even point, fragile stability
1.1	−9.2	−1.8	−8.4	Stable	Moderate profitability, system stability
1.2	−9.2	−3.6	−8.4	Stable	High profitability, but may trigger excessive competition

#### 4.4.3 Impact of rewards and punishments

Based on the sensitivity analysis results of parameters *M* and *F*, this study reveals that the government incentive-punishment mechanism exhibits significant threshold effects and synergistic effects. Numerical simulation results presented in Table 8 indicate that the critical value for the reward parameter *M* is approximately 1.8, while that for the punishment parameter *F* is approximately 7.2. Below these thresholds, the system cannot escape the initial non-cooperative equilibrium state (0, 0, 0). When *M*≥2 and *F*≥8, the system can stably converge to the ideal cooperative equilibrium point (1, 1, 0), achieving a market-based cooperative stable state. Furthermore, the synergistic configuration of the M and F parameters decisively influences the system's evolutionary speed. Under the baseline configuration (*M* = 2, *F* = 8), the convergence time is approximately 28 cycles, whereas under the fast-start configuration (*M* = 2.5, *F* = 10), it can be reduced to 20 cycles. Notably, diminishing marginal returns emerge when *M*>4 or *F*>16, indicating that excessive incentive penalties not only increase government fiscal burdens but may also reduce market efficiency. Therefore, policymakers should dynamically adjust the M-F parameter combination according to different developmental stages of HMW recovery governance. We propose: initially employ stronger incentives (*M* = 2.5 − 2 = 3.5, *F* = 8 − 10) to rapidly establish a cooperative mechanism. During the mature phase, intervention intensity can be appropriately reduced (*M* = 1.5 − 2, *F* = 6 − 8) to optimize the cost-benefit ratio. This finding provides quantitative evidence for governments to construct adaptive reward-punishment governance systems.

**Table 8:** Impact of rewards and punishments.

Parameter conditions	System status	Theoretical significance
*M* < 1.8, *F* < 7.2	(0, 0, 0)	Policy measures are insufficient
*M*≈1.8, *F*≈7.2	state transition begins	Policy effects are beginning to take hold
*M*≥2, *F*≥8	(1, 1, 0)	The ideal state of reward-punishment governance
*M* = 2, *F* = 8	Convergence time 20	Optimal cost-benefit state
*M* = 2.5, *F* = 10	Convergence time 25	The strong incentive governance

Through the above analysis, we identify a crucial strategic evolution from (0, 0, 0) to (1, 1, 0) requires a three-phase approach:

(1) Enhance residents perceived benefits through environmental education. Clearly designate government leadership (e.g., environmental authorities collaborating with communities) to conduct HMW risk awareness campaigns. Utilize engaging formats such as short video releases, social media check-ins, and offline prize quizzes to encourage broader resident participation. Set quantifiable targets, such as raising resident awareness from 30 to 50%. Budget allocation should reference the *W* = 0.5 parameter calibration result, e.g., 5 USD per capita annually.
(2) Increase the profitability of medical waste disposal institutions. The government can boost institution's profit margins within 1–3 years by implementing “subsidies + tax breaks,” referencing *R*′/*I* = 1.1. Simultaneously, it can establish a “tiered pricing mechanism based on recycling volume,” where higher recycling volumes command higher disposal fees per unit to prevent institution's negative response.
(3) Control government regulatory costs to avoid market mechanism disruption. As overall environmental awareness rises across society, governments can gradually reduce regulatory intensity and begin establishing “resident oversight” mechanisms. For instance, by enabling residents to evaluate medical waste disposal institutions' services through an app, with evaluation results linked to institutions' subsidies, regulatory costs *D* can be reduced to less than 50% of the original level.

This approach respects evolutionary laws, enables smooth system guidance, emphasizes cost-effectiveness with fiscal sustainability, and provides scientific, operational pathways for environmental governance.

## 5 Discussion and conclusion

### 5.1 Discussion

This study constructs an evolutionary game model involving community residents, medical waste disposal institutions, through theoretical analysis, model analysis and numerical simulation. Specifically, the study finds the following:

First, from an evolutionary stable strategy perspective, the equilibrium (1, 1, 0) representing compliant recycling practices by residents and institutions under loose government regulation that under loose government regulation, constitutes the system's evolutionary stable equilibrium. Numerical simulations reveal three negative eigenvalues, confirming local stability. More importantly, the system converges to this equilibrium regardless of the initial conditions, demonstrating sustained attractiveness and global stability. By contrast, the deal comprehensive cooperation (1, 1, 1) state lacks stability within the evolutionary game framework. This counterintuitive result can be explained by the concept of diminishing marginal returns of regulatory intervention. When the government simultaneously bears regulatory costs, incentive expenditures, and educational investments, marginal regulatory costs eventually exceed marginal social benefits, creating institutional inefficiency. This finding challenges the conventional wisdom that stricter government regulation necessarily yields better outcomes (38), whose models assumed fully rational actors and excluded social interactions. Instead, we show excessive intervention disrupts the self-organizing capacity of residents and institutions, and social norms can partially substitute for formal regulation, reducing the need for sustained government oversight. This discrepancy highlights the added value of integrating behavioral mechanisms into evolutionary governance models.

Second, peer effects play a crucial role in HMW recycling system evolution. When the resident compliant recycling proportion exceeds the critical threshold of 50%, compliance costs fall below non-compliance costs, creating positive social norm effects. The substantial difference in eigenvalues on the two sides of the peer-effect threshold demonstrates a significant shift in residents' compliance behavior. This provides quantitative evidence for the robustness of the peer effect hypothesis, and reveals the mechanism by which peer effects influence community residents' compliance with HMW recycling by affecting endogenous psychological costs. This mechanism aligns with the peer influence framework proposed by Manski (11), where endogenous effects (group behavior shaping individual behavior) dominate once a critical mass is reached. We extend the work of Pinheiro and Vasconcelos (19), by situating peer-effect and threshold effects of general collective behavior within a tripartite governance context. This finding provides micro-theoretical evidence for fostering residents' voluntary compliance with recycling, and offers policymakers a reference for evaluating investments in public environmental education.

Third, we quantify the threshold values at which key policy parameters become effective. Numerical analysis reveals that: (1) environmental education investment exhibits a leap effect, with the system skipping intermediate stages to reach optimal equilibrium directly when investment exceeds *W*>0.5; (2) institutional profitability follows a moderate principle, with stability requiring the profitability ratio to remain within 1.05–1.15, while excessive profits (>1.15) trigger opportunistic behavior; and (3) government rewards and punishments display significant threshold effects, with convergence to optimal cooperation requiring minimum thresholds of *M*≥2 and *F*≥8, beyond which diminishing marginal returns emerge. These threshold-based policy insights resonate with recent policy experiments. For instance, California's Drug Takeback Solutions Foundation penalty case (39) illustrates how exceeding moderate profit thresholds triggers opportunistic behavior.

In view of the above, we present and validate a three-phase incremental evolution strategy: (1) enhancing residents perceived benefits through environmental education; (2) increasing the profitability of medical waste disposal institutions; (3) controlling government regulatory costs to avoid market mechanism disruption. This approach respects evolutionary laws, achieves smooth transition from non-cooperation (0, 0, 0) to the stable state (1, 1, 0), emphasizes cost-effectiveness with fiscal sustainability, and provides scientific, operational pathways for promote a healthy living environment and wellbeing.

### 5.2 Conclusion

This study advances the theoretical understanding of HMW recycling governance by integrating evolutionary game theory with peer effect. Unlike prior studies that treat economic incentives and social norms as separate mechanisms, our framework captures their dynamic interaction, provides a novel analytical framework for complex environmental governance, and enriches evolutionary game theory applications in environmental governance. Theoretically, we contribute to the literature by (1) constructing a comprehensive tripartite evolutionary game framework, the incentive and constraint mechanisms of government policies, the behavioral interaction mechanisms of community residents, and the strategic influence mechanisms of medical waste disposal institutions, thereby filling the one-dimensional research perspective of existing research; (2) integrating dynamic evolutionary game model with peer effects, not only moves beyond static cross-sectional designs to reveal how new norms form, diffuse, and evolve among multiple stakeholders, but also simulates the dynamic process and threshold through which policy design triggers such diffusion; (3) filling the theoretical gap in household medical waste management.

From a policy design perspective, our three-phase progressive evolutionary strategy carries significant methodological implications. This strategy abandons traditional one-step linear thinking, adopting gradual adjustment that respects system evolutionary laws. This evolutionary stability-based policy design paradigm offers new approaches to complex social problem governance with strong generalizability. Additionally, this study supports a “limited but effective” government regulation approach, and reveals that excessive intervention may backfire, suggesting a transformation from controlling government to guiding government.

For medical waste disposal institutions, our results emphasize the importance of market mechanism cultivation. The stability of (1, 1, 0) indicates that spontaneous resident-institution cooperation proves more sustainable than comprehensive tripartite cooperation. This suggests focusing on stimulating and maintaining market actors' intrinsic motivations rather than relying solely on external constraints through price mechanism improvement, access system optimization, and information disclosure strengthening.

### 5.3 Research limitations

This study has several limitations. regarding model assumptions, the study inadequately considers heterogeneity characteristics among participants. In reality, residents' environmental awareness varies significantly across regions, medical waste disposal institutions differ in scale and technology, and government fiscal capacity and governance preferences vary considerably. Such heterogeneity may affect system evolutionary paths and equilibrium outcomes. Future research should introduce heterogeneity parameters, constructing more realistic analytical models. Regarding exogenous dynamic mechanisms, this study primarily focuses on endogenous evolutionary forces with insufficient consideration of exogenous shocks. In reality, technological progress, policy environment changes, and emergencies significantly impact system evolution. Future research should introduce exogenous shock variables, analyzing system response mechanisms and adaptive capacity to external disturbances. Regarding parameter estimation, this study's parameter setting lacks large-scale empirical data support. Despite sensitivity analysis, parameter accuracy requires verification. Future research should strengthen field investigation and data collection through surveys, interviews, and experimental studies to obtain more precise parameter estimates.

## Data Availability

The original contributions presented in the study are included in the article/supplementary material, further inquiries can be directed to the corresponding author.
